# Influence of the Cognitive and Emotional Status of Patients with Chronic Pain on Treatment Success (Reduction in Pain Intensity and Adherence to Pharmacotherapy): A Prospective Study

**DOI:** 10.3390/ijerph192315968

**Published:** 2022-11-30

**Authors:** Dijana Hnatešen, Ivan Radoš, Iva Dimitrijević, Dino Budrovac, Ivan Omrčen, Roman Pavić, Ivana Gusar, Maja Čebohin, Krešimir Šolić

**Affiliations:** 1 Faculty of Medicine Osijek, Josip Juraj Strossmayer University of Osijek, 31000 Osijek, Croatia; 2 Nursing Institute “Professor Radivoje Radić”, Faculty of Dental Medicine and Health Osijek, Josip Juraj Strossmayer University of Osijek, 31000 Osijek, Croatia; 3 Clinical Department of Pain Management, University Hospital Osijek, 31000 Osijek, Croatia; 4 Clinical Hospital of Traumatology, University Hospital Centre “Sestre Milosrdnice”, 10000 Zagreb, Croatia; 5 Department of Health Studies, University of Zadar, 23000 Zadar, Croatia; 6 Medical School Osijek, 31000 Osijek, Croatia; 7 Faculty of Electrical Engineering, Computer Science and Information Technology Osijek, Josip Juraj Strossmayer University of Osijek, 31000 Osijek, Croatia

**Keywords:** emotional status, cognitive status, chronic pain, adherence to pharmacotherapy, pain reduction

## Abstract

This prospective study aimed to determine the cognitive and emotional status among patients with chronic pain and to examine the potential influence on the treatment success, measured by the reduction in pain intensity and adherence to pharmacotherapy. A total of seventy patients were followed for two months. The results of the comparison between patients who did and did not follow the physician’s instructions regarding adherence to pharmacotherapy showed a significant difference in cognitive status and a reduction in pain intensity. Patients who followed the physician’s instructions on taking analgesics had significantly higher scores on the Montreal Cognitive Assessment (MoCA) of cognitive status and a substantially higher reduction in pain intensity. Scores on the MoCA test provide statistically significant indications regarding patients’ decision to follow instructions regarding adherence to pharmacotherapy. Scores on the MoCA test, anxiety, age, and pain intensity (measured with a numeric rating scale—NRS) on admission were identified as potential predictors for the reduction in pain intensity. The linear regression model was statistically significant (*χ*^2^ = 40.0, *p* < 0.001), explained between 43.5% and 61.1% of variance regarding the reduction in pain intensity. The findings of this study show that cognitive status, measured with MoCA, and emotional status, measured with the Depression, Anxiety, and Stress Scale (DASS-21), significantly influence the reduction in pain intensity and adherence to pharmacotherapy. The results suggest that cognitive and emotional status may be potential predictors of treatment success. This finding points to the importance of a biopsychosocial approach in the treatment of chronic pain, where an important emphasis can be placed on the psychosocial determinants of pain.

## 1. Introduction

The treatment of chronic pain (CP) requires a multimodal and multidisciplinary approach, where pharmacotherapy and analgesics are cornerstones in treatment [1,2,3]. Chronic pain presents a severe social burden, being a significant global health issue [4,5] that affects one in five people worldwide [6], and is associated with a complex interplay of physical, emotional, mental, and spiritual challenges that influence each other and result in poor function, reduced quality of life, and stigma [7,8]. Moreover, chronic pain bears a high economic cost [9]. CP is defined by the International Association for the Study of Pain as persistent pain, regardless of normal tissue healing, for three months or more [10]. CP involves a complex and variable interplay between biological, psychological, and social factors. It is categorized by the World Health Organization (WHO) as a chronic disease because it may last a lifetime, can lead to functional impairment, is irreversible, and requires patient rehabilitation, regular medical care, and supervision [11]. Chronic non-malignant pain (CNMP) prevalence estimates greatly vary, ranging from 8% to 60% in the general population [12]. Moreover, pain has been described as an unpleasant sensory or emotional experience, and when experiencing pain consciously, cognitive processing becomes imperative. For a better understanding, it is necessary to state that cognition is defined as the brain’s ability to acquire, process, store, and retrieve information.

Moreover, pain evaluation strongly depends on cognition, as it requires learning and the recall of previous experiences [13]. Patients seeking treatment for chronic pain often report difficulties with cognitive functioning, and a wide range of causes may lead to reports of cognitive deficits. They may be a product of a primary neurological disorder, medications, the pain itself, preoccupation with injuries or illness, sleep deprivation, stressful circumstances, some other emotional state, or a combination of these factors [14]. Any cognitive deficit can impact a person’s ability to adhere to prescribed medication, which can lead to medication errors, medication-related hospital admissions, and dependence on others to assist with medication management tasks [15,16]. Despite the effectiveness of pain medicines, recent data suggest that approximately 40% of prescription medicines are not taken as prescribed [17]. Moreover, among adults with chronic illnesses such as diabetes or hypertension, between 30% and 50% of medications are not taken as prescribed [18,19]. According World Health Organization (WHO) adherence has been defined as “the extent to which a person’s behavior—taking medication, following a diet, and/or executing lifestyle changes—corresponds with agreed recommendations from a healthcare provider”. The definition implies that the patient is an active partner of the healthcare professional, rather than a sole recipient accepting medical instructions, and refers to responsibilities for both. Adherence is challenging, especially for chronic disease conditions. Non-adherence to medication may lead to a worsening disease and increased mortality and has been considered a global problem of striking magnitude [20]. Poor adherence is associated with increased morbidity and mortality. It may account for approximately 125,000 deaths and 10% of hospitalizations in the United States annually [17], leading to the worsening of the disease and a substantial rise in healthcare costs [21]. There are many possible factors related to pharmacotherapy that may influence non-adherence, such as the complexity of the therapeutic regimen, lack of efficacy, side effects, duration of treatment, concomitant treatment regimens, various changes in prescriptions during treatment, immediate availability of the healthcare professionals, and the associated economic costs [22]. Moreover, the relationships between healthcare providers and patients may affect adherence [23]. Non-adherence to medication is conceptualized, giving the reported taxonomy as, “the process by which patients do not take their medications as prescribed” [24], and it can occur in three distinct, interrelated phases (initiation, implementation, and persistence) according to the European Society for Patient Adherence, Compliance, and Persistence [25]. Permanent interactions between physical (pain), psychological (emotions and cognition), and social factors are affected by significant individual differences in patients experiencing disability due to chronic pain [26]. Research has shown that depression and anxiety worsen chronic pain, interfere with treatment, and have a negative impact on the quality of life of people with a higher intensity and longer duration of pain, reduced perception of control, use of passive coping strategies, and intense behavioral changes [27,28]. The biopsychosocial model is a widely accepted approach to understanding the important factors influencing the experience of chronic pain.

Therefore, this study aimed to determine the cognitive and emotional status regarding the level of anxiety, depression, and stress among patients with chronic pain and to examine the potential influence on the treatment success, measured by the reduction in pain intensity and adherence to pharmacotherapy.

## 2. Materials and Methods

### 2.1. Study Design

**Figure 1:**
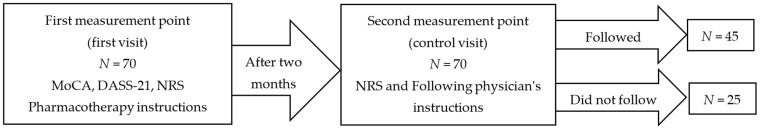
Study design.

The study was conducted at the Department of Pain Management, University Hospital Centre Osijek, over two months, from June to the end of August 2021. At the beginning of the study, the patients had their first physician’s examination (i.e., first measurement point) to assess the intensity of pain on a numerical rating scale (NRS) and received instructions by the physicians for further treatment and pharmacotherapy. They were then referred to a psychologist for a brief assessment of cognitive status using the Montreal Cognitive Assessment (MoCA) and of anxiety, depression, and stress using the Depression, Anxiety, and Stress Scale (DASS-21). At the control physician’s examination (i.e., second measurement point), pain intensity was reassessed and adherence (“followed”, “did not follow”) to the physician’s instructions on taking the prescribed analgesics was assessed (Figure 1).

### 2.2. Patients

All patients who attended their first physician’s examination (i.e., measurement point) in this period were included. Inclusion criteria were: Chronic non-malignant pain (pain ≥ 3 months), >18 years old, who understood the Croatian language and script, and voluntary participation in the study. According to the pain diagnosis, 39 patients with low back pain, 14 patients with cervical pain, 9 patients with joint pain, 4 patients with headache, 3 patients with trigeminal neuralgia, and 1 patient with diabetic polyneuropathy were identified from the total sample.

### 2.3. Outcome Measure

#### 2.3.1. Numeric Rating Scale (NRS)

For the assessment of the intensity of pain, a numeric rating scale (NRS) was used (0—no pain, 10—strongest possible pain), which is a widely used screening instrument [29]. The NRS is short, easy to administer, correlates with other measures of pain intensity, and is used in many large healthcare systems [30]. Based on previous studies and clinical practice, we categorized pain screening NRS scores as mild (1–3), moderate (4–6), or severe (7–10). Studies of chronic pain patients with different conditions have reached varying conclusions about the optimal cut-off points for mild, moderate, and severe pain on the 0–10 NRS, with 4 or 5 being the most commonly recommended lower limits for moderate pain and 7 or 8 for severe pain [31,32,33]. Participants at the first visit were asked what their average pain intensity was in the last three months, and at the control visit they were asked what their average pain was in the last two months.

#### 2.3.2. The Montreal Cognitive Assessment (MoCA)

For the assessment of the patient’s cognitive status, the Montreal Cognitive Assessment (MoCA) test was used. The MoCA is a brief screening tool for cognitive impairment, scored from 0 to 30. It is a one-page protocol that assesses eight cognitive domains (attention, executive function, calculation, language, working memory and recall, abstraction, orientation, and visuospatial processing) [34]. The originally suggested cut-off for the diagnosis of cognitive impairment (CI) was a score of below 26 [35]. The cut-off score of 18 is usually considered to separate mild cognitive impairment from Alzheimer’s disease (AD). However, there is an overlap in the scores since, by definition, AD is determined by the presence of cognitive impairment in addition to the loss of autonomy. The average MoCA score for mild cognitive impairment (MCI) is 22 (range 19–25), and the average MoCA score for mild AD is 16 [36]. There is evidence supporting that MoCA is a reliable test for cognitive status in chronic patients [37].

#### 2.3.3. The Depression, Anxiety, and Stress Scale (DASS-21)

For the assessment of the patients’ emotional status, the Depression, Anxiety, and Stress Scale (DASS-21) was used. The Depression, Anxiety, and Stress Scale is a measure of self-assessment that examines the frequency and severity of negative emotional states of depression, anxiety, and stress over the past seven days. The scale consists of 3 measures of associated negative affective states classified into 3 subscales, with a total of 21 items [38]. The patient responds to each item by expressing how often they have experienced the condition described in the statement in the past week, rounding off one of the answers on a four-point Likert-type scale. The answers range from 0 (not applicable to me at all) to 3 (almost entirely or most of the time related to me). The total score is obtained by summing the rounded values with the range of scores of each subscale from 0 to 21. A higher overall score on the subscales shows more intense symptoms of depression, anxiety, and stress. The results obtained on the subscales are interpreted in accordance with the cut-off results for the agreed symptom severity marks, classified into five levels for each subscale, separately. There is evidence supporting that DASS-21 subscales demonstrate adequate measurement properties for research involving groups with chronic pain [39].

#### 2.3.4. Adherence to the Physician’s Instructions

For the assessment of the adherence to pharmacotherapy, physicians asked the participants at the control examination (second measurement point) whether they took the pharmacotherapy medication according to the physician’s instructions for a period of two months. Physicians recorded answers as “followed” or “did not follow” in medical documentation.

### 2.4. Statistical Methods

For statistical analysis, we used standard statistical methods. All collected categorical data are presented with absolute and relative frequencies, while numerical data are presented with median, interquartile range, and total range, distributed within parameters that did not follow a normal Gaussian distribution. Differences between two independent sets of numerical data were tested with the nonparametric Mann–Whitney U test, while differences between two dependent sets of numerical data were tested with the nonparametric Wilcoxon test. Differences between categorical data were tested with the Chi-square test. Exploratory logistic regression and multiple regression were used to identify potential predictors regarding adherence to pharmacotherapy and reductions in pain intensity. The strength of the relationship between pairs of measurements was tested with the nonparametric Spearman’s correlation test or Kendall’s Tau correlation test. The level of statistical significance was set at α = 0.05. Statistical data analysis was performed with MedCalc^®^ statistical software, version 20.026 (MedCalc Software Ltd., Ostend, Belgium; https://www.medcalc.org (accessed on 10 October 2021).

### 2.5. Ethical Consideration

This study was approved by the Ethics Committee (R2-13170/2020) of the authors’ institution, following the principles of the 1983 revised Helsinki Declaration guiding research on human subjects. All patients gave their written informed consent for participation in the study.

## 3. Results

This study included 70 patients. Demographic characteristics, adherence to pharmacotherapy, MoCA, and DASS-21 scores are shown in Table 1.

**Figure 2:**
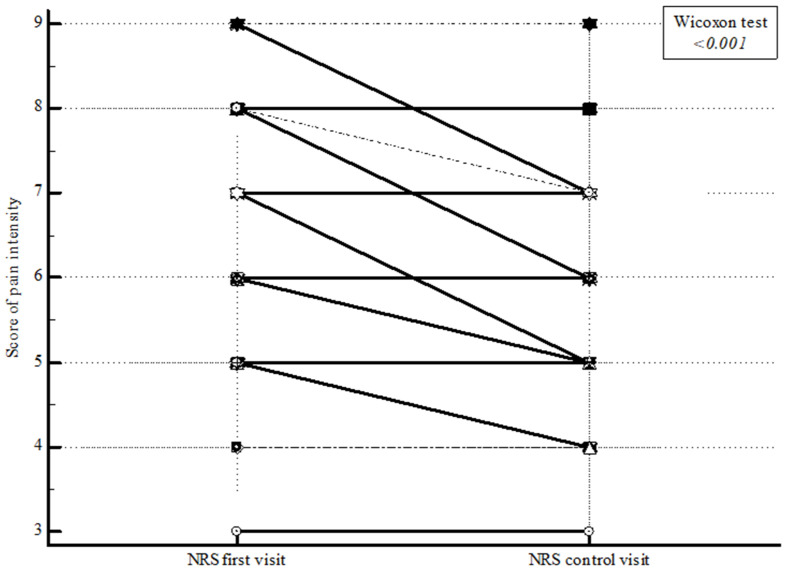
The reduction of pain intensity.

In 20.0% of patients, a decrease in pain intensity was noted for one score, while in 11.4% it was noted for two scores at the control examination after two months (second measurement point) (Figure 2).

The results of the comparison between patients regarding adherence to pharmacotherapy showed a significant difference in cognitive status and the reduction in pain intensity. Patients who followed physicians’ instructions on taking analgesics had a significantly higher score on the MoCA assessment of cognitive status (Mann–Whitney test, *p* < 0.001) and a significantly higher reduction in pain intensity (Mann–Whitney test, *p* = 0.04) compared to those who did not follow the physician’s instructions. There was no significant difference found regarding gender (Chi-square test, *p* = 0.24), NRS at first visit (Mann–Whitney test, *p* = 0.13), NRS at control visit (Mann–Whitney test, *p* = 0.56), or DASS-21 depression (Mann–Whitney test, *p* = 0.40), anxiety (Mann–Whitney test, *p* = 0.32), and stress (Mann–Whitney test, *p* = 0.54) subscale scores (Table 2).

Scores on the MoCA test made statistically significant contributions to patients’ decisions to follow instructions regarding adherence to pharmacotherapy (Hosmer–Lemeshow test, *p* = 0.71). The model was statistically significant (*χ*^2^ = 23.4, *p* = 0.002), explained between 28.4% (Cox and Snell) and 39.0% (Negelkerke) of variance regarding adherence to pharmacotherapy, and correctly classified 75.7% of all cases (Table 3). ROC analysis for the MoCA test yielded a cut-off value of 24, with sensitivity of 68.9% and specificity of 80.0% (Youden index 0.49, AUC = 0.80, *p* < 0.001). However, age was not identified as a significant contributor in this model, even though there was a significant difference in age between patients who followed instructions and patients who did not (Table 2). Age and scores on the MoCA test had a high correlation coefficient (Spearman’s correlation test, rho = −0.64, *p* < 0.001) and age highly influenced the scores on the MoCA test, with R^2^ = 0.338 (least squares regression; *F*-ratio, *p* < 0.001).

A positive, low, and statistically significant correlation of the reduction in pain intensity was found with age (Tau = 0.24, *p* = 0.004) and NRS at the baseline visit (Tau = 0.23, *p* = 0.006), while a negative, moderate, statistically significant correlation was found with the anxiety DASS-21 score (Tau = −0.21, *p* = 0.01). There was no correlation found between the reduction in pain intensity and cognitive status, depression, or stress DASS-21 score (Table 4). Moreover, there was no difference between genders regarding the reduction in pain intensity.

In the following logistic regression model, 22 (31.4%) patients had a reduction in pain intensity. Scores on the MoCA test, patient’s age, and level of anxiety made statistically significant contributions to the reduction in pain intensity (Hosmer–Lemeshow test, *p* = 0.36). The model was statistically significant (*χ*^2^ = 40.0, *p* < 0.001), explained between 43.5% (Cox and Snell) and 61.1% (Negelkerke) of variance regarding the reduction in pain intensity, and correctly classified 82.9% of all cases (Table 5).

There was no significant correlation found between the MoCA test and each DASS-21 test, or between patients’ ages and each DASS-21 test. However, there was a significant, moderate, positive correlation between NRS at first visit and DASS-21, for both anxiety (Spearman’s correlation test, rho = 0.27, *p* = 0.02) and depression (Spearman’s correlation test, rho = 0.35, *p* = 0.003).

## 4. Discussion

This study’s results showed that cognitive and emotional status significantly influenced patients’ reduction in pain intensity and adherence to pharmacotherapy. Therefore, the results suggest that cognitive and emotional status may be potential predictors of chronic pain treatment success. In this study, we decided to measure the success of treatment via the reduction in pain intensity and adherence to pharmacotherapy.

The cognitive status among patients with chronic pain was most often in the category of mild cognitive impairment. The emotional status assessment has shown that patients with chronic pain most often have scores for depression and anxiety in the mild category, while stress was in the normal category. A significant finding of this study can be recognized by the significant difference in success on the cognitive assessment test between patients who followed the physician’s instructions related to taking pharmacotherapy and those who did not follow those instructions. There was a significant difference between patients who followed the physician’s instructions and patients who did not follow the instructions regarding the cognitive assessment, i.e., patients who followed the instructions had better cognitive abilities. Moreover, patients who followed the instructions improved significantly (statistically more significant difference) in terms of pain intensity. Nevertheless, the logistic regression analysis suggests an interesting result. Even patients who did not consistently follow the physician’s instructions showed a decrease in pain intensity. There are probably some factors that were not identified by this research which may have an influence on the reduction of pain intensity. Additionally, it may be necessary to consider that the research was performed with a small sample. Therefore, this result should be taken as preliminary data. Observing the age variable, studies indicate that older adults show a lower level of adherence [40,41], which was confirmed by the results of this study. It is necessary to emphasize that age was not associated with adherence to treatment after controlling for other factors. Older people probably suffer pain for a longer period of time, which could also affect their cognitive status and likelihood of following instructions. It was noted that older people follow instructions less frequently, so there is probably an influence of age on cognitive ability. This finding is consistent with the literature, showing that cognitive impairment challenges the ability to adhere to the complex medication regimens needed to treat multiple medical problems in older adults [15,42]. Moreover, Dufton, Iezzi et al. and McGuire showed that most patients with chronic pain reported poor memory and concentration in their daily activities [43,44,45,46]. However, in the logistic regression model, only cognitive status was detected as a potential predictor, with OR equal to 1.33 (while, at the same time, age has OR 1.0). We can assume that in patients with a lower score on the cognitive assessment scale, there was a higher risk that they would not follow the physician’s instructions related to taking pharmacotherapy.

In this study, the results suggest a significant pain reduction. This result was significantly negatively correlated with anxiety and positively with patients’ age, but not correlated with mental status. However, the logistic regression model has identified mental status as a potential predictor for pain intensity reduction. Shuchang et al. showed that comorbidities such as depression and anxiety could impact pain perception [47]. Research by Roth et al. showed that cognitive complaints were associated with depression, fatigue, female sex, and pain catastrophizing in patients with chronic pain [48]. Furthermore, McCracken and Iverson showed that the use of antidepressants, pain severity, pain-related anxiety, and depression were associated with cognitive complaints, and that depression accounted for the largest unique proportion of variance in cognitive complaints [14].

Additionally, our results demonstrate a significant positive correlation between pain intensity at first visit and depression and anxiety. The conclusions of several studies indicate that depression and anxiety are predictors of worsening chronic pain over time and that this relationship is reciprocal [27]. Due to the poor emotional state caused by depression and anxiety, we can assume that patients are less likely to follow the physician’s instructions. This could be supported by the literature, which states that, among other aspects, emotions, vulnerability, self-compassion, and motivation commonly impact chronic pain patients [49]. This anxiety often leads to the avoidance of activities that can exacerbate pain and worsen disability [50]. In this study, results showed that high-intensity stress affects patients’ ability to follow physicians’ instructions, which could explain why more pain causes more stress. In a desire to reduce pain and stress, patients agree to follow instructions. Research by Abdullah and Geha even suggests that chronic stress and chronic pain syndromes are “two sides of the same coin” and highlight the limbic system’s central role and learning mechanisms in maintaining chronic stress and chronic pain [51]. Nevertheless, anxiety and stress can actually increase pain perception and reduce pain coping skills [52]. Although the literature states that pain and stress are both adaptive in protecting the organism, if either of the two processes becomes chronic, this can lead to long-term “maladaptive” changes in physiology, and consequently behavior, resulting in suffering and compromised well-being [53]. Nevertheless, Thomas and Lee noted that “zero pain is not the goal”, but reducing pain is also important [54]. The results of this study are consistent with earlier research that also reported a small magnitude of pain reduction [54,55]. Namely, according to Qaseem et al., improvement in activities of daily living and health-related quality of life should be the ultimate goal [55]. However, it is necessary to measure the intensity of pain. Even a lower pain intensity can consequently negatively affect the health-related quality of life and lead to a poorer emotional status [8]. The findings of this study are consistent with Bruns and Disorbio. They proposed that psychological evaluation should be an integral part of the diagnostic workup for chronic pain because of prodigious evidence favoring the parallel relationship between chronic pain and mental health [56].

## 5. Limitations

The limitations of this study were the small sample size, the predominant older age group, the lack of data on the type of prescribed analgesics, and the study being conducted in one health institution. Therefore, the plan is to repeat this study on a larger sample with a control group to examine cause-and-effect findings. Additionally, we can investigate which interventions would help patients with cognitive deficits to more easily follow their physician’s instructions about taking pharmacotherapy. This is a significant issue because a large number of patients with chronic pain are members of the elderly population, with many other comorbidities. Furthermore, another limitation was the impossibility of objectively verifying the patients’ answers regarding compliance with the instructions received from the physicians. In the following study, it will be necessary to include a standardized questionnaire to assess adherence. Lastly, it is important not to ignore the fact that all questionnaires have biases.

## 6. Implications for Practice

Despite some limitations, the results of this study highlight the importance of procedures that reduce the risk of the improper use of pharmacotherapy. Considering that cognitive status is a potential predictor of adherence, by identifying it, we have the possibility to influence adherence by adjusting interventions.

## 7. Conclusions

The findings of this study showed that cognitive status measured with the MoCA, and emotional status measured with the Depression, Anxiety, and Stress Scale (DASS-21), significantly influenced the reduction in pain intensity and adherence to pharmacotherapy among patients. The results suggest that cognitive and emotional status may be potential predictors of treatment success. This finding points to the importance of a biopsychosocial approach in the treatment of chronic pain, where an important emphasis can be placed on the psychosocial determinants of pain.

## Figures and Tables

**Table 1:** Distribution of patients according to demographic characteristics, adherence, MoCA, and DASS-21 scores.

Variables	Categories	No. (%)	*p*
Gender	Male	26 (37.1)	0.06 *
	Female	44 (62.9)	
MoCA test	Normal cognitive functioning	32 (45.7)	0.03 *
	Mild cognitive impairment (MCI)	28 (40.0)	
	Dementia (AD)	10 (14.3)	
Adherence to pharmacotherapy	Followed	45 (64.3)	0.09 *
	Did not follow	25 (35.7)	
	Categories	Me (25–75%)	*p*
NRS	First measurement point	6.0 (5.0 to 8.0)	<0.001 ^†^
	Second measurement point	5.0 (5.0 to 7.0)	
	Me	(25–75%)	Min–Max
Age	59.0	51.0 to 72.0	29.0 to 80.0
MoCA test	25.0	22.0 to 26.0	14.0 to 29.0
Depression DASS-21	5.0	1.0 to 8.0	0 to 21.0
Anxiety DASS-21	5.0	1.0 to 11.0	0 to 20.0
Stress DASS-21	6.0	4.0 to 8.0	0 to 21.0

* Chi-square test; ^†^ Wilcoxon test (with Hodges–Lehmann median difference of −0.5).

**Table 2:** Comparison between patients regarding adherence to pharmacotherapy.

Variables	Followed Instructions	Did Not Follow	*p*
	*n* = 45	*n* = 25	
	No. (%)		
Male	19 (42.2)	7 (28.0)	0.24 *
Female	26 (57.8)	18 (72.0)	
	Me (25–75%)		
Age	53.0 (49.0 to 68.0)	70.0 (57.3 to 74.5)	0.01 ^†^
NRS first visit	6.0 (5.0 to 8.0)	5.0 (5.0 to 7.0)	0.13 ^†^
NRS control visit	5.0 (5.0 to 7.0)	5.0 (5.0 to 6.3)	0.56 ^†^
Reduction in pain intensity	0 (0 to 1.0)	0 (0 to 0)	0.04 ^†^
MoCA test	26.0 (23.0 to 27.0)	22.0 (16.0 to 24.0)	<0.001 ^†^
Depression DASS-21	5.0 (1.0 to 7.0)	6.0 (1.8 to 8.0)	0.40 ^†^
Anxiety DASS-21	4.0 (1.0 to 9.0)	6.0 (2.0 to 12.0)	0.32 ^†^
Stress DASS-21	6.0 (4.0 to 8.0)	4.0 (3.0 to 12.0)	0.54 ^†^

* Chi-square test; ^†^ Mann–Whitney test.

**Table 3:** Prediction model regarding adherence to pharmacotherapy.

Predictor	β	Wald	*p* *	OR	95% CI of OR	
					Lower	Upper
MoCA	0.28	6.17	0.01	1.33	1.06	1.66
Age	−0.002	0.004	0.95	1.0	0.93	1.07
NRS first visit	0.32	1.18	0.28	1.38	0.77	2.49
Anxiety DASS-21	−0.11	1.34	0.25	0.89	0.74	1.08
Depression DASS-21	−0.11	0.54	0.46	0.89	0.66	1.21
Stress DASS-21	0.17	0.92	0.34	1.18	0.84	1.66
Male gender	0.11	0.02	0.88	1.11	0.28	4.36
Constant	−7.64	3.77	0.05			

* Logistic regression analysis with Enter method, variable contributes significantly to the prediction.

**Table 4:** Correlation between reduction in pain intensity and cognitive status, emotional status (depression, anxiety, and stress), age, gender, NRS at first visit, and NRS at control visit.

Matching Variables		Tau	95% CI	*p*
Reduction in pain intensity	MoCA test	0.003	−0.18 to 0.19	0.98 *
	Depression DASS-21	0.007	−0.16 to 0.17	0.93 *
	Anxiety DASS-21	−0.21	−0.36 to −0.04	0.01 *
	Stress DASS-21	−0.04	−0.21 to 0.13	0.59 *
	Age	0.24	0.06 to 0.39	0.004 *
	NRS first measurement point	0.23	0.03 to 0.40	0.006 *
	Gender	Me (25–75%)		
		Male	Female	
		*n* = 26	*n* = 44	
		0 (0 to 1.0)	0 (0 to 1.0)	0.49 ^†^

* Kendall’s Tau correlation test; ^†^ Mann–Whitney test.

**Table 5:** Prediction model regarding reduction in pain intensity.

Predictor	β	Wald	*p* *	OR	95% CI of OR	
					Lower	Upper
MoCA	0.42	5.14	0.02	1.53	1.06	2.21
Age	0.24	6.96	0.008	1.28	1.06	1.53
NRS first visit	0.08	0.04	0.83	1.08	0.53	2.17
Anxiety DASS-21	−0.78	6.54	0.01	0.46	0.25	0.83
Depression DASS-21	0.03	0.03	0.87	1.03	0.69	1.54
Stress DASS-21	0.67	2.67	0.10	1.96	0.87	4.39
Did not follow instructions	−1.08	1.16	0.28	0.34	0.05	2.42
Male gender	1.20	1.21	0.27	3.32	0.39	28.15
Constant	−26.35	8.59	0.003			

* Logistic regression analysis with Enter method, variable contributes significantly to the pre-diction.

## Data Availability

The data presented in this study are available upon request from the corresponding author.
